# A reference genome assembly of the declining tricolored blackbird, *Agelaius tricolor*

**DOI:** 10.1093/jhered/esac053

**Published:** 2022-09-13

**Authors:** Kimberly M Ballare, Merly Escalona, Kelly Barr, William Seligmann, Samuel Sacco, Ruta Madhusudan Sahasrabudhe, Oanh Nguyen, Christy Wyckoff, Thomas B Smith, Beth Shapiro

**Affiliations:** Department of Ecology and Evolutionary Biology, University of California Santa Cruz, Santa Cruz, CA, United States; Department of Biomolecular Engineering, University of California Santa Cruz, Santa Cruz, CA, United States; Department of Ecology and Evolutionary Biology, University of California Los Angeles, Los Angeles, CA, United States; Center for Tropical Research, Institute of the Environment and Sustainability, University of California, Los Angeles, Los Angeles, CA, United States; Department of Ecology and Evolutionary Biology, University of California Santa Cruz, Santa Cruz, CA, United States; Department of Ecology and Evolutionary Biology, University of California Santa Cruz, Santa Cruz, CA, United States; UC Davis Genome Center, DNA Technologies and Expression Analysis Cores, University of California, Davis, Davis, CA, United States; UC Davis Genome Center, DNA Technologies and Expression Analysis Cores, University of California, Davis, Davis, CA, United States; Santa Lucia Conservancy, Carmel, CA, United States; Department of Ecology and Evolutionary Biology, University of California Los Angeles, Los Angeles, CA, United States; Center for Tropical Research, Institute of the Environment and Sustainability, University of California, Los Angeles, Los Angeles, CA, United States; Department of Ecology and Evolutionary Biology, University of California Santa Cruz, Santa Cruz, CA, United States; Howard Hughes Medical Institute, University of California Santa Cruz, Santa Cruz, CA, United States

**Keywords:** California Conservation Genomics Project, CCGP, Icteridae, long read assembly, new world blackbirds, whole genome

## Abstract

The tricolored blackbird, *Agelaius tricolor*, is a gregarious species that forms enormous breeding and foraging colonies in wetland and agricultural habitats, primarily in California, USA. Once extremely abundant, species numbers have declined dramatically in the past century, largely due to losses of breeding and foraging habitats. Tricolored blackbirds are currently listed as Endangered by the IUCN, and Threatened under the California Endangered Species Act. Increased genetic information is needed to detail the evolutionary consequences of a species-wide bottleneck and inform conservation management. Here, we present a contiguous tricolored blackbird reference genome, assembled with PacBio HiFi long reads and Dovetail Omni-C data to generate a scaffold-level assembly containing multiple chromosome-length scaffolds. This genome adds a valuable resource for important evolutionary and conservation research on tricolored blackbirds and related species.

## Introduction

**Fig. 1.**
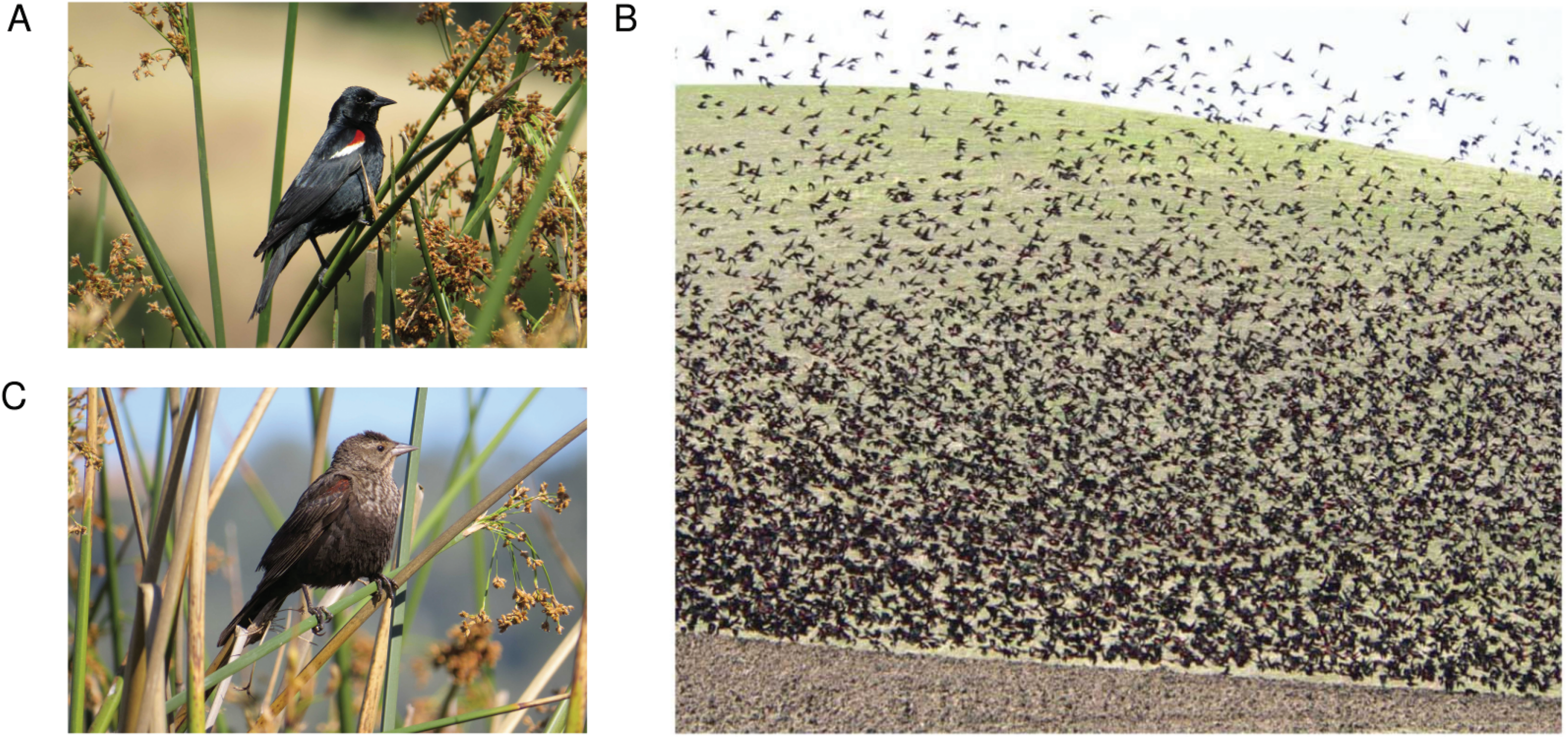
Tricolored blackbirds, *Agelaius tricolor*, (A) adult male © 2014 Christy Wycoff, (B) large tricolored blackbird flock © 2007 Robert Meese and (C) adult female © 2014 Christy Wycoff.

The tricolored blackbird (*Agelaius tricolor*, Audubon 1837) is a charismatic member of the Icteridae, the new world blackbirds. It is considered near-endemic to California, USA, with over 99% of the population occurring within the state (Graves et al. 2013). Its current range also extends into Baja California, Mexico and includes small breeding populations in Oregon, Washington, and Nevada, USA. “Tricoloreds” are similar in appearance to their abundant and widespread sister taxon, the red-winged blackbird (*A. pheoniceus*), with males displaying a bright red and white epaulet on each wing (Fig. 1A). Unlike red-winged blackbirds, which are highly territorial, tricoloreds are gregarious and non-territorial, forming the largest breeding colonies of any extant North American passerine since the extinction of the passenger pigeon (*Ectopistes migratorius*) (Fig. 1B).

Tricolored blackbird populations have declined precipitously over the past century. Once considered the most abundant bird in California, biologists documented over a 60% decline in abundance from the 1930s to the 1980s, and by another 45% from 2008 to 2017 (Graves et al. 2013; Meese 2017). The primary cause of these declines has been the loss and degradation of breeding and foraging habitat in wetlands (Dahl 1990). As a consequence, the majority of tricoloreds have shifted from nesting primarily in native marshes (Neff 1937) to croplands, predominantly triticale (a rye-wheat hybrid mainly grown for cattle feed, Beedy et al. 2020). However, this resulted in lower reproductive success largely due to 1) severely reduced insect prey in nearby foraging habitat (Meese 2013), and 2) crop harvest during critical nesting periods, where tens of thousands of nests could be destroyed in a single day (Beedy and Hamilton 1997). Additionally, recent surveys suggest that some critically important colony sites in previous triticale fields have now been converted to perennial nut orchards and vineyards, which are not viable tricolored habitats (Beedy et al. 2020 and references therein). Tricolored blackbirds are considered endangered by the IUCN (2011), and were recently assigned threatened status under the California Endangered Species Act (2019). The species is protected at the U.S. federal level under the Migratory Bird Treaty Act, but has not yet been assigned a conservation status through the U.S. Federal Endangered Species Act.

There is currently limited genetic knowledge of tricoloreds. A recent reduced genome representation dataset (RAD-Seq) suggests extant populations are panmictic, with high gene flow across the species range and little evidence for selection (Barr et al. 2021). Two marker-based studies have produced somewhat conflicting results, showing low levels of genetic diversity and high levels of inbreeding in specific populations (Berg et al. 2010; Liu and Meese 2020). Given the severe recent species-wide bottleneck and range-wide habitat fragmentation, increased genetic information from whole genomes is needed to fully understand the past and current status of gene flow, selection, and other evolutionary processes.

Here, we present a highly contiguous and complete de novo genome assembly for the tricolored blackbird, generated from a wild-caught female individual using Pacific Biosciences (PacBio, CA) HiFi long reads and Omni-C data. The final genome is 1.15 Gb across 213 scaffolds, with a scaffold N50 of 47.7 Mb and a BUSCO score of 97.2%. This genome is one of the first high-quality genomes generated for the bird family Icteridae, which contains several threatened and endangered species. The tricolored blackbird genome was generated as part of a larger effort to assemble numerous genomes for diverse taxa across the state of California (The California Conservation Genomics Project—CCGP; Shaffer et al. 2022). This reference genome will enable evolutionary research of tricolored blackbirds and relatives, helping inform conservation management efforts for this dynamic species persisting in rapidly changing landscapes.

## Methods

### Biological materials

We collected blood from a wild female tricolored blackbird captured near Carmel-by-the-Sea, CA, USA, roughly the north–south midpoint of their geographic range. The sample was collected on 10 May 2020 using a baited walk-in trap to capture the individual (collection permits: CA SCP—13085 and USGS BBL Master Bander—24154). We determined sex via morphological characteristics (Fig. 1C), choosing a female for the genome assembly as the heterogametic sex. We obtained approximately 100 µL of blood by jugular vein puncture (Sheldon et al. 2008), then monitored and released the individual after sample collection. The blood sample was then stored in EDTA buffer at −20 °C until DNA extraction. Note that precise capture location information is withheld due to conservation concerns of a threatened and endangered species. Please contact the corresponding author if more information is required.

### Nucleic acid extraction, library preparation, and sequencing

#### High molecular weight genomic DNA isolation

High molecular weight (HMW) genomic DNA (gDNA) was isolated from whole blood preserved in EDTA. 20 µL of whole blood was added to 2 ml of lysis buffer containing 10 mM Tris–HCl pH 8.0, 25 mM EDTA, 0.5% (w/v) SDS, and 100 µg/ml Proteinase K. Lysis was carried out at room temperature for a few hours until the solution was homogenous. The lysate was treated with 20 µg/ml RNase A at 37 °C for 30 min and cleaned with equal volumes of phenol/chloroform using phase lock gels (Quantabio, MA; Cat. # 2302830). DNA was precipitated by adding 0.4× volume of 5 M ammonium acetate and 3× volume of ice cold ethanol. The DNA pellet was washed twice with 70% ethanol and resuspended in an elution buffer (10 mM Tris, pH 8.0). Purity of gDNA was accessed using NanoDrop ND-1000 spectrophotometer and 260/280 ratio of 1.83 and 260/230 ratios of 2.34 was observed. DNA yield (120 µg total) was quantified using Qbit 2.0 Fluorometer (Thermo Fisher Scientific, MA). Integrity of the HMW gDNA was verified on a Femto pulse system (Agilent Technologies, CA).

### PacBio HiFi library preparation

The HiFi SMRTbell library was constructed using the SMRTbell Express Template Prep Kit v2.0 (PacBio, Cat. #100-938-900) according to the manufacturer’s instructions. HMW gDNA was sheared to a target DNA size distribution between 15 and 20 kb. The sheared gDNA was concentrated using 0.45× of AMPure PB beads (PacBio, Cat. #100-265-900) for the removal of single-strand overhangs at 37 °C for 15 min, followed by further enzymatic steps of DNA damage repair at 37 °C for 30 min, end repair and A-tailing at 20 °C for 10 min and 65 °C for 30 min, ligation of overhang adapter v3 at 20 °C for 60 min and 65 °C for 10 min to inactivate the ligase, then nuclease treated at 37 °C for 1 h. The SMRTbell library was purified and concentrated with 0.45× Ampure PB beads (PacBio, Cat. #100-265-900) for size selection using the BluePippin/PippinHT system (Sage Science, MA; Cat. #BLF7510/HPE7510) to collect fragments greater than 7 to 9 kb. The 15 to 20 kb average HiFi SMRTbell library was sequenced at University of California Davis DNA Technologies Core (Davis, CA) using 2 8M SMRT cells, Sequel II sequencing chemistry 2.0, and 30-h movies each on a PacBio Sequel II sequencer.

### Omni-C library preparation

The Omni-C library was prepared using Dovetail Omni-C Kit (Dovetail Genomics, CA) according to the manufacturer’s protocol with slight modifications. Briefly, chromatin was fixed in place in the nucleus. Fixed chromatin was digested with DNase I then extracted. Chromatin ends were repaired and ligated to a biotinylated bridge adapter followed by proximity ligation of adapter containing ends. After proximity ligation, crosslinks were reversed and the DNA purified from proteins. Purified DNA was treated to remove biotin that was not internal to ligated fragments. An NGS library was generated using an NEB Ultra II DNA Library Prep kit (New England Biolabs, MA) with an Illumina compatible y-adaptor. Biotin-containing fragments were then captured using streptavidin beads. The post capture product was split into 2 replicates prior to PCR enrichment to preserve library complexity with each replicate receiving unique dual indices. The library was sequenced at Vincent J. Coates Genomics Sequencing Lab (Berkeley, CA) on an Illumina NovaSeq platform (Illumina, CA) to generate approximately 100 million 2 × 150 bp read pairs per GB genome size.

### Nuclear genome assembly

We assembled the tricolored blackbird genome following the CCGP assembly protocol Version 4.0 (outlined in Table 1, see Data availability statement for link to all assembly scripts). This protocol uses PacBio HiFi reads and Omni-C data for the generation of high-quality and highly contiguous nuclear genome assemblies while minimizing manual curation. We removed remnant adapter sequences from the PacBio HiFi dataset using HiFiAdapterFilt (Sim et al. 2022) and obtained the initial dual or partially phased diploid assembly (http://lh3.github.io/2021/10/10/introducing-dual-assembly) using HiFiasm with the filtered PacBio HiFi reads and the Omni-C dataset (Cheng et al. 2021). We tagged output haplotype 1 as the primary assembly, and output haplotype 2 as the alternate assembly. We identified sequences corresponding to haplotypic duplications, contig overlaps, and repeats on the primary assembly with purge_dups (Guan et al. 2020) and transferred them to the alternate assembly. We scaffolded both assemblies using the Omni-C data with SALSA (Ghurye et al. 2017, 2019).

**Table 1.** Assembly pipeline and software used.

Assembly	Software and options^a^	Version
Filtering PacBio HiFi adapters	HiFiAdapterFilt	Commit 64d1c7b
K-mer counting	Meryl (k = 21)	1
Estimation of genome size and heterozygosity	GenomeScope	2
De novo assembly (contiging)	HiFiasm (Hi-C Mode, –primary, output p_ctg.hap1, p_ctg.hap2)	0.16.1-r375
Remove low-coverage, duplicated contigs	purge_dups	1.2.5
**Scaffolding**		
Omni-C scaffolding	SALSA (-DNASE, -i 20, -p yes)	2
Gap closing	YAGCloser (-mins 2 -f 20 -mcc 2 -prt 0.25 -eft 0.2 -pld 0.2)	Commit 0e34c3b
**Omni-C contact map generation**		
Short-read alignment	BWA-MEM (-5SP)	0.7.17-r1188
SAM/BAM processing	samtools	1.11
SAM/BAM filtering	pairtools	0.3.0
Pairs indexing	pairix	0.3.7
Matrix generation	cooler	0.8.10
Matrix balancing	hicExplorer (hicCorrectmatrix correct --filterThreshold -2 4)	3.6
Contact map visualization	HiGlass	2.1.11
	PretextMap	0.1.4
	PretextView	0.1.5
	PretextSnapshot	0.0.3
**Organelle assembly**		
Mitogenome assembly	MitoHiFi (-r, -p 50, -o 1)	2 commit c06ed3e
**Genome quality assessment**		
Basic assembly metrics	QUAST (--est-ref-size)	5.0.2
Assembly completeness	BUSCO (-m geno, -l aves)	5.0.0
	Merqury	2020-01-29
**Contamination screening**		
Local alignment tool	BLAST+	2.10
General contamination screening	BlobToolKit	2.3.3
**Comparison to *A. phoeniceus***		
Sequence alignment	nucmer (mummer)	4.0.0rc1
Nucmer output to Dot! preparation	DotPrep.py (--overview 10000) https://github.com/marianattestad/dot	Commit b18fed0
Dot plot visualization	DOT	Last accessed 8 September 2020

Software citations are listed in the text.

^a^Options detailed for non-default parameters.

We generated Omni-C contact maps for both assemblies by aligning the Omni-C data against the corresponding assembly with BWA-MEM (Li 2013), identified ligation junctions, and generated Omni-C pairs using pairtools (Goloborodko et al. 2018). We generated a multiresolution Omni-C matrix with cooler (Abdennur and Mirny 2020) and balanced it with hicExplorer (Ramírez et al. 2018). We used HiGlass (Kerpedjiev et al. 2018) and the PretextSuite (https://github.com/wtsi-hpag/PretextView; https://github.com/wtsi-hpag/PretextMap; https://github.com/wtsi-hpag/PretextSnapshot) to visualize the contact maps. We checked the contact maps for major misassemblies. We cut the assemblies at the closest joins where the misassemblies were found. No further joins were made after this step. Using the PacBio HiFi reads and YAGCloser (https://github.com/merlyescalona/yagcloser), we closed some of the remaining gaps generated during scaffolding. We then checked for contamination using the BlobToolKit Framework (Challis et al. 2020). Finally, we trimmed remnants of sequence adaptors and mitochondrial contamination identified during NCBI contamination screening.

### Mitochondrial genome assembly

We assembled the mitochondrial genome of the tricolored blackbird from the PacBio HiFi reads using the reference-guided pipeline MitoHiFi (https://github.com/marcelauliano/MitoHiFi; Uliano-Silva et al. 2021). The mitochondrial sequence of *Agelaius phoeniceus* (NCBI:MN356439.1) was used as the starting reference sequence. After completion of the nuclear genome, we searched for matches of the resulting mitochondrial assembly sequence in the nuclear genome assembly using BLAST+ (Camacho et al. 2009) and filtered out contigs and scaffolds from the nuclear genome with a percentage of sequence identity >99% and size smaller than the mitochondrial assembly sequence.

### Genome size estimation and quality assessment

We generated k-mer counts from the PacBio HiFi reads using meryl (https://github.com/marbl/meryl). The k-mer database was then used in GenomeScope 2.0 (Ranallo-Benavidez et al. 2020) to estimate genome features including genome size, heterozygosity, and repeat content. To obtain general contiguity metrics, we ran QUAST [Version 5.0.2] (Gurevich et al. 2013). To evaluate genome quality and completeness we used BUSCO [Version 5.0.0] (Manni et al. 2021) with the Aves ortholog database (aves_odb10) which contains 8,338 genes. Assessment of base level accuracy (QV) and k-mer completeness was performed using the previously generated meryl database and merqury (Rhie et al. 2020). We further estimated genome assembly accuracy via BUSCO gene set frameshift analysis using the pipeline described in Korlach et al. (2017). Measurements of the size of the phased blocks are based on the size of the contigs generated by HiFiasm on HiC mode (initial diploid assembly). We follow the quality metrics nomenclature established by Rhie et al. (2020), we will use the derived genome quality notation *x*·*y*·*P*·*Q*·*C*, where *x* = log10[contig NG50]; *y* = log10[scaffold NG50]; *P* = log10[phased block NG50]; *Q* = Phred base accuracy QV (quality value); *C* = % genome represented by the first “*n*” scaffolds, following a karyotype of 2*n* = 80 inferred from ancestral taxa, *A. phoeniceus* (Bird Chromosome Database V3.0/2022 [https://sites.unipampa.edu.br/birdchromosomedatabase/chromosome-number-in-birds/]; The Animal Chromosome Count Database Release 1.0.1 [https://cromanpa94.github.io/ACC/, 03/24/21]). Quality metrics for the notation were calculated on the primary assembly.

### Comparison to *A. phoeniceus* genome assembly

To obtain draft chromosome assignments, we aligned our genome (bAgeTri1.0.p) to the annotated genome assembly for the red-winged blackbird *A. phoeniceus* (Brood Parasite Genomics Consortium, GenBank Accession GCA_020745825.1) using nucmer (Marçais et al. 2018). We then used the default settings in Dot (https://dot.sandbox.bio/) to visualize forward, reverse, and repetitive alignments across the 2 genomes.

## Results

**Fig. 2.**
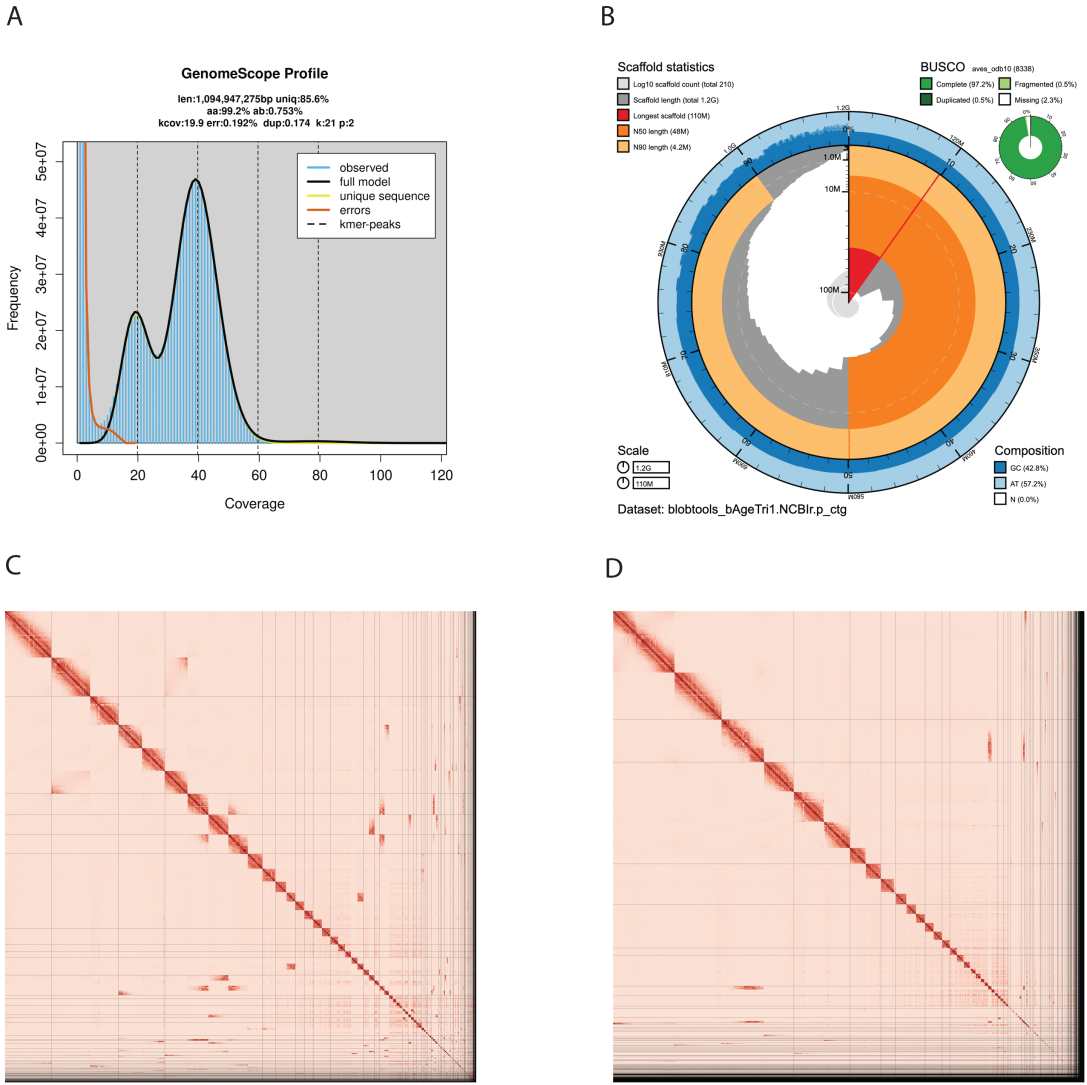
Visual overview of genome assembly metrics. (A) K-mer spectrum output generated from PacBio HiFi data without adapters using GenomeScope 2.0. The bimodal pattern observed corresponds to a diploid genome. K-mers covered at lower coverage and lower frequency correspond to differences between haplotypes, and the higher coverage and higher frequency k-mers correspond to the similarities between haplotypes. (B) BlobToolKit Snail plot showing a graphical representation of the quality metrics presented in Table 2 for the *Agelaius tricolor* primary assembly (bAgeTri1.0.p). The plot circle represents the full size of the assembly. From the inside-out, the central plot covers length-related metrics. The red line represents the size of the longest scaffold; all other scaffolds are arranged in size order moving clockwise around the plot and drawn in gray starting from the outside of the central plot. Dark and light orange arcs show the scaffold N50 and scaffold N90 values. The central light gray spiral shows the cumulative scaffold count with a white line at each order of magnitude. White regions in this area reflect the proportion of Ns in the assembly The dark versus light blue area around it shows mean, maximum and minimum GC versus AT content at 0.1% intervals (Challis et al. 2020). Omni-C Contact maps for the primary (C) and alternate (D) genome assembly generated with PretextSnapshot. Hi-C contact maps translate proximity of genomic regions in 3D space to contiguous linear organization. Each cell in the contact map corresponds to sequencing data supporting the linkage (or join) between 2 of such regions. Scaffolds are separated by black lines and higher density corresponds to higher levels of fragmentation.

The Omni-C and PacBio HiFi sequencing libraries generated 69.3 million read pairs and 2.7 million reads, respectively. The latter yielded 41.39-fold coverage (N50 read length 16,691 bp; minimum read length 48 bp; mean read length 16,549 bp; maximum read length of 56,219 bp) based on the GenomeScope 2.0 genome size estimation of 1.09 Gb. Based on PacBio HiFi reads, we estimated 0.192 % sequencing error rate and 0.756% nucleotide heterozygosity rate. The k-mer spectrum based on PacBio HiFi reads shows (Fig. 2A) a bimodal distribution with 2 major peaks at 19- and 39-fold coverage, where peaks correspond to homozygous and heterozygous states of a diploid species. The distribution presented in this k-mer spectrum supports that of a low heterozygosity profile.

The final assembly (bAgeTri1) consists of 2 pseudo haplotypes, primary and alternate, both genome sizes similar to the estimated value from GenomeScope 2.0 (Fig. 2A). The primary assembly consists of 214 scaffolds spanning 1.15 Gb with contig N50 of 22.6 Mb, scaffold N50 of 47.7 Mb, longest contig of 95.9 Mb and largest scaffold of 113.9 Mb. On the other hand, the alternate assembly consists of 450 scaffolds, spanning 1.14 Gb with contig N50 of 21.9 Mb, scaffold N50 of 63.4 Mb, largest contig 96.1 Mb and largest scaffold of 149.1 Mb. Detailed assembly statistics are reported in tabular form in Table 2, and graphical representation for the primary assembly in Fig. 2B (see Supplementary Fig. 1 for the alternate assembly). The primary assembly has a BUSCO completeness score of 97.2% using the Aves gene set, a per-base quality (QV) of 65.9, a k-mer completeness of 91 and a frameshift indel QV of 41.75; while the alternate assembly has a BUSCO completeness score of 92.8% using the same gene set, a per-base quality (QV) of 65.1, a k-mer completeness of 84 and a frameshift indel QV of 41.79.

**Table 2.** Sequencing and assembly statistics, and accession numbers.

BioProjects and vouchers	CCGP NCBI BioProject			PRJNA720569			
	Genera NCBI BioProject			PRJNA766258			
	Species NCBI BioProject			PRJNA808324			
	NCBI BioSample			SAMN25872474			
	Specimen identification			1412-34295			
	NCBI Genome accessions			Primary		Alternate	
	Assembly accession			JALJCS000000000		JALJCT000000000	
	Genome sequences			GCA_023055355.1		GCA_023055385.1	
Genome sequence	PacBio HiFi reads	Run		1 PACBIO_SMRT (Sequel II) run: 2.7M spots, 45.3G bases, 29.7 Gb			
		Accession		SRX15223267			
	Omni-C Illumina reads	Run		2 ILLUMINA (Illumina NovaSeq 6000) runs: 69.4M spots, 19G bases, 6.1 Gb			
		Accession		SRX15223268, SRX15223269			
Genome Assembly Quality Metrics	Assembly identifier (quality code^a^)				bAgeTri1(7.7.P7.Q65.C89)		
	HiFi read coverage^b^			41.39×			
				Primary		Alternate	
	Number of contigs			311		547	
	Contig N50 (bp)			22,601,135		21,939,630	
	Contig NG50 (bp)^b^			26,514,841		25,737,790	
	Longest contigs			95,987,212		96,155,385	
	Number of scaffolds			214		450	
	Scaffold N50 (bp)			47,739,980		63,409,879	
	Scaffold NG50 (bp)^b^			48,798,145		63,409,879	
	Largest scaffold			113,901,592		149,100,169	
	Size of final assembly (bp)			1,157,608,456		1,142,379,013	
	Phased block NG50 (bp)			26,514,841		25,737,790	
	Gaps per Gbp (# Gaps)			84 (97)		85 (97)	
	Indel QV (frameshift)			41.75		41.79	
	Base pair QV			65.9486		65.1717	
				Full assembly = 65.5454			
	K-mer completeness			91.2971		84.4351	
				Full assembly = 99.5636			
	BUSCO completeness (aves), *n* = 8,338		C	S	D	F	M
		P^c^	97.20%	96.70%	0.50%	0.50%	2.30%
		A^c^	92.80%	92.20%	0.60%	0.60%	6.60%
	Organelles			1 complete mitochondrial sequence CM041028.1			

^a^Assembly quality code *x*·*y*·*P*·*Q*·*C* derived notation, from (Rhie et al. 2021). *x* = log10[contig NG50]; *y* = log10[scaffold NG50]; *P* = log10[phased block NG50]; *Q* = Phred base accuracy QV (quality value); *C* = % genome represented by the first “*n*” scaffolds, following a known karyotype for blackbirds of 2*n* = 80 (inferred from ancestral taxa). Quality code for all the assembly denoted by primary assembly (bAgeTri1.0.p). BUSCO scores. (C)omplete and (S)ingle; (C)omplete and (D)uplicated; (F)ragmented and (M)issing BUSCO genes. *n*, number of BUSCO genes in the set/database.

^b^Read coverage and NGx statistics have been calculated based on the estimated genome size of 1.09 Gb.

^c^P(rimary) and A(lternate) assembly values.

We identified 1 misassembly and broke a single join made by SALSA on the primary assembly. We were able to close a total of 6 gaps, 4 on the primary assembly and 2 on the alternate. Finally, we filtered out a single contig from the primary assembly corresponding to mitochondrial contamination. No further contigs were removed. The Omni-C contact maps show that both assemblies are highly contiguous, and although not chromosome level, they both contain multiple chromosome-length scaffolds (Fig. 2C and D).

We observed that all of the *A. phoeniceus* chromosomes had forward alignments with at least 1 scaffold in the *A. tricolor* genome (Supplementary Fig. 2), with at least 20 chromosome-length scaffolds recovered. We detail the specific chromosome-scaffold alignments in Supplementary Table 1.

We assembled a mitochondrial genome with MitoHiFi. Final mitochondrial genome size was 16,775 bp. The base composition of the final assembly version is A = 30.62%, C = 32.05%, G = 13.93%, T = 23.4%, and consists of 22 unique transfer RNAs and 13 protein coding genes.

## Discussion

Here, we present a complete draft genome assembly of the tricolored blackbird, assembled using long reads and chromosome-scale sequencing data. This assembly is 2 orders of magnitude more contiguous than a previous de novo genome assembled with short-read data (Barr et al. 2021). There are currently 4 species in the Icteridae with genomes available on NCBI. The tricolored blackbird genome has the fewest scaffolds of any Icterid genome currently available, less than half the scaffold number for the other 2 chromosome-level assemblies in the family (Supplementary Table 2). Other than *A. tricolor*, there are several other threatened or endangered species in the Icteridae including the congener *A. xanthomus*, the yellow shouldered blackbird, which do not have genomic information currently available. This genome will add a valuable resource to existing Icterid genomes for important evolutionary research on tricolored blackbirds and other related species.

High-resolution genomic data will also enable vital research to inform species-specific conservation efforts of tricolored blackbirds. First, it will allow us to resolve currently conflicting results from marker-based genetic studies, and assess contemporary patterns of gene flow and genetic structure across the entire tricoloreds’ range. Additionally, this genome will enable comparisons of modern population genetic structure and diversity with pre-1940’s populations using museum specimens. Because DNA sequenced from curated specimens is usually severely fragmented, a highly continuous reference genome will allow for more accurate genotype calling and analysis of historic populations. With these data, we can assess genetic diversity both before and after a species-wide bottleneck, investigating the consequence of key evolutionary processes. Despite dramatic and ongoing species-wide declines, tricoloreds have yet to be assigned endangered status under the federal Endangered Species Act, but is currently listed as a Species of Conservation Concern (USFWS 2008), as well as defined as Threatened under the California Endangered Species Act. Increased genetic information will inform conservation management by helping to define population genetic units, identify potential isolated populations, and quantify levels of genetic diversity.

## Supplementary Material

esac053_suppl_Supplementary_Figure_S1

esac053_suppl_Supplementary_Figure_S2

esac053_suppl_Supplementary_Tables

## Data Availability

Data generated for this study are available under NCBI BioProject PRJNA808324. Raw sequencing data for sample 1412-34295 (NCBI BioSample SAMN25872474) are deposited in the NCBI Short Read Archive (SRA) under SRX15223267 for PacBio HiFi sequencing data, and SRX15223268, SRX15223269 for the Omni-C Illumina sequencing data. GenBank accessions for both primary and alternate assemblies are GCA_023055355.1 and GCA_023055385.1; and for genome sequences JALJCS000000000 and JALJCT000000000. The GenBank organelle genome assembly for the mitochondrial genome is CM041028.1. Assembly scripts and other data for the analyses presented can be found at the following GitHub repository: www.github.com/ccgproject/ccgp_assembly.
