# Supplementary material for: A reference genome assembly of the declining tricolored blackbird, Agelaius tricolor
Source: J Hered. 2022 Sep 13;114(1):44–51. doi: 10.1093/jhered/esac053 (PMC10019024; doi:10.1093/jhered/esac053)

Supplementary Figure 2. Visualization of whole-genome alignment between *A. tricolor* genome assembly (bAgeTri1.0.p) and *A. phoeniceus* (NCBI Bioproject PRJNA735624, GenBank Accession GCA_020745825.1). For *A. tricolor* scaffold alignments to all assembled chromosomes in the *A. phoeniceus* genome see Supplementary Table 1.

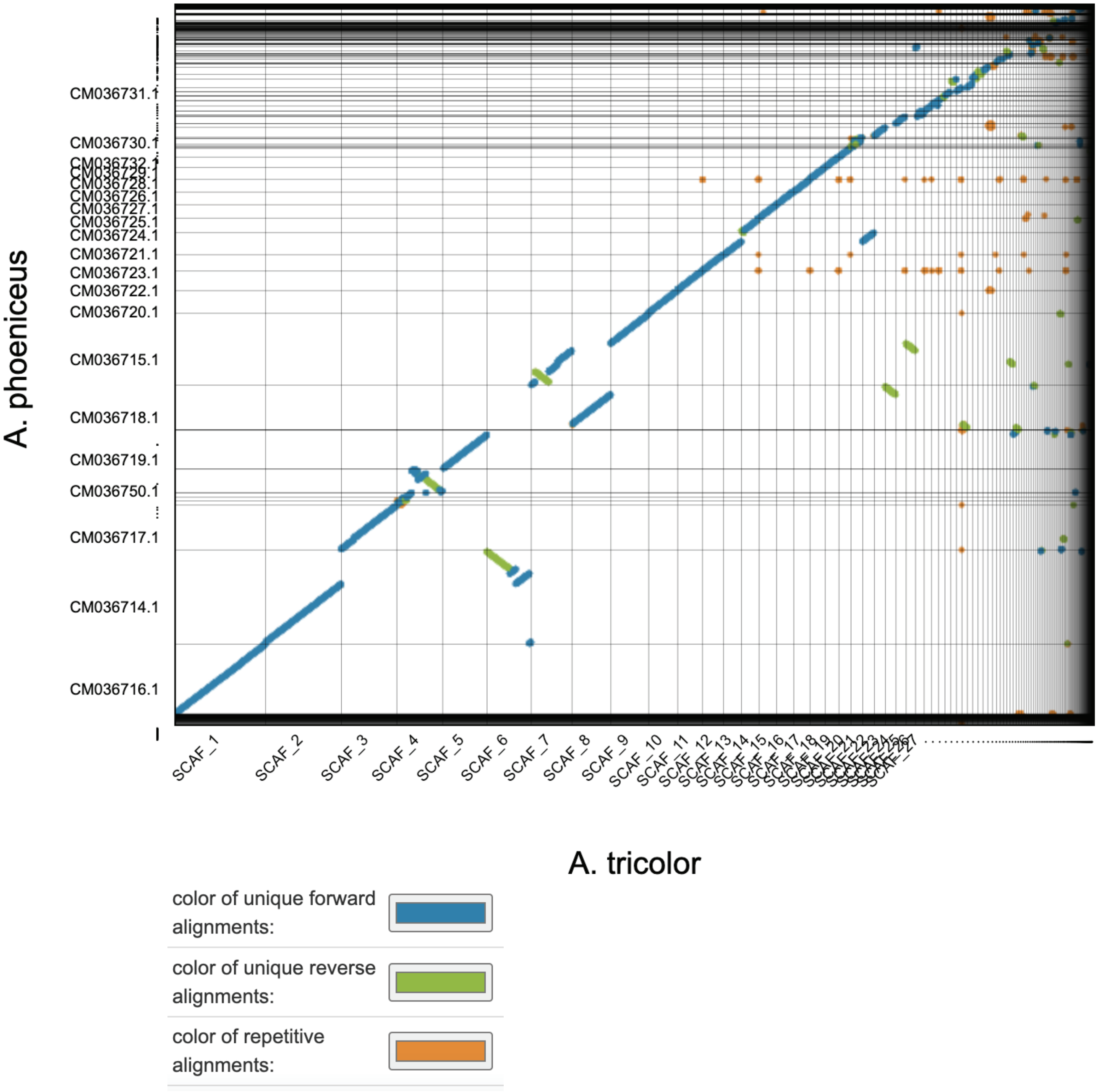

Supplement: esac053_suppl_Supplementary_Figure_S2 [file esac053_suppl_supplementary_figure_s2.pdf]
