# Supplementary material for: A reference genome assembly of the declining tricolored blackbird, Agelaius tricolor
Source: J Hered. 2022 Sep 13;114(1):44–51. doi: 10.1093/jhered/esac053 (PMC10019024; doi:10.1093/jhered/esac053)
Supplement: esac053_suppl_Supplementary_Tables [file esac053_suppl_supplementary_tables.docx]

**Supplementary Table 1.** Chromosome alignment from Dot (https://github.com/marianattestad/dot) comparing *A. tricolor* (bAgeTri1.0.p) with *A. phoeniceus* (GCA_020745825.1, NCBI Bioproject PRJNA735624).

| *A. phoeniceus* Chromosome Number | *A. phoeniceus* Chromosome NCBI ID/Link | *A. tricolor* aligned scaffold number(s) |
| --- | --- | --- |
| 1 | [CM036714.1](https://www.ncbi.nlm.nih.gov/nuccore/CM036714.1) | SCAF_2, SCAF_6, SCAF_58, SCAF_103 |
| 2 | [CM036715.1](https://www.ncbi.nlm.nih.gov/nuccore/CM036715.1) | SCAF_7, SCAF_9, SCAF_26, SCAF_45, SCAF_71, SCAF_79, SCAF_143 |
| 3 | [CM036716.1](https://www.ncbi.nlm.nih.gov/nuccore/CM036716.1) | SCAF_1 |
| 4 | [CM036717.1](https://www.ncbi.nlm.nih.gov/nuccore/CM036717.1) | SCAF_3, SCAF_72, SCAF_74, SCAF_86 |
| 5 | [CM036718.1](https://www.ncbi.nlm.nih.gov/nuccore/CM036718.1) | SCAF_8, SCAF_24, SCAF_34, SCAF_47, SCAF_54, |
| 6 | [CM036719.1](https://www.ncbi.nlm.nih.gov/nuccore/CM036719.1) | SCAF_5, SCAF_46, SCAF_62, SCAF_67, SCAF_82, SCAF_108 |
| 7 | [CM036720.1](https://www.ncbi.nlm.nih.gov/nuccore/CM036720.1) | SCAF_10 |
| 8 | [CM036721.1](https://www.ncbi.nlm.nih.gov/nuccore/CM036721.1) | SCAF_13, SCAF_22 |
| 9 | [CM036722.1](https://www.ncbi.nlm.nih.gov/nuccore/CM036722.1) | SCAF_11, SCAF_39 |
| 10 | [CM036723.1](https://www.ncbi.nlm.nih.gov/nuccore/CM036723.1) | SCAF_12, SCAF_12 |
| 11 | [CM036724.1](https://www.ncbi.nlm.nih.gov/nuccore/CM036724.1) | SCAF_14, SCAF_93 |
| 12 | [CM036725.1](https://www.ncbi.nlm.nih.gov/nuccore/CM036725.1) | SCAF_15 |
| 13 | [CM036726.1](https://www.ncbi.nlm.nih.gov/nuccore/CM036726.1) | SCAF_17 |
| 14 | [CM036727.1](https://www.ncbi.nlm.nih.gov/nuccore/CM036727.1) | SCAF_16 |
| 15 | [CM036728.1](https://www.ncbi.nlm.nih.gov/nuccore/CM036728.1) | SCAF_18, SCAF_42 |
| 16 | [CM036729.1](https://www.ncbi.nlm.nih.gov/nuccore/CM036729.1) | SCAF_19 |
| 17 | [CM036730.1](https://www.ncbi.nlm.nih.gov/nuccore/CM036730.1) | SCAF_23, SCAF_49 |
| 18 | [CM036731.1](https://www.ncbi.nlm.nih.gov/nuccore/CM036731.1) | SCAF_32, SCAF_35 |
| 19 | [CM036732.1](https://www.ncbi.nlm.nih.gov/nuccore/CM036732.1) | SCAF_20 |
| 20 | [CM036733.1](https://www.ncbi.nlm.nih.gov/nuccore/CM036733.1) | SCAF_37, SCAF_38 |
| 21 | [CM036734.1](https://www.ncbi.nlm.nih.gov/nuccore/CM036734.1) | SCAF_25 |
| 22 | [CM036735.1](https://www.ncbi.nlm.nih.gov/nuccore/CM036735.1) | SCAF_29 |
| 23 | [CM036736.1](https://www.ncbi.nlm.nih.gov/nuccore/CM036736.1) | SCAF_28 |
| 24 | [CM036737.1](https://www.ncbi.nlm.nih.gov/nuccore/CM036737.1) | SCAF_36 |
| 25 | [CM036738.1](https://www.ncbi.nlm.nih.gov/nuccore/CM036738.1) | SCAF_30 |
| 26 | [CM036739.1](https://www.ncbi.nlm.nih.gov/nuccore/CM036739.1) | SCAF_51 |
| 27 | [CM036740.1](https://www.ncbi.nlm.nih.gov/nuccore/CM036740.1) | SCAF_31 |
| 28 | [CM036741.1](https://www.ncbi.nlm.nih.gov/nuccore/CM036741.1) | SCAF_33 |
| 29 | [CM036742.1](https://www.ncbi.nlm.nih.gov/nuccore/CM036742.1) | SCAF_21, SCAF_27, SCAF_46 |
| 30 | [CM036743.1](https://www.ncbi.nlm.nih.gov/nuccore/CM036743.1) | SCAF_41, SCAF_70 |
| 31 | [CM036744.1](https://www.ncbi.nlm.nih.gov/nuccore/CM036744.1) | SCAF_40 |
| 32 | [CM036745.1](https://www.ncbi.nlm.nih.gov/nuccore/CM036745.1) | SCAF_57 |
| 33 | [CM036746.1](https://www.ncbi.nlm.nih.gov/nuccore/CM036746.1) | SCAF_66, SCAF_69 |
| 34 | [CM036747.1](https://www.ncbi.nlm.nih.gov/nuccore/CM036747.1) | SCAF_42, SCAF_42 |
| 35 | [CM036748.1](https://www.ncbi.nlm.nih.gov/nuccore/CM036748.1) | SCAF_11, SCAF_43 |
| W | [CM036749.1](https://www.ncbi.nlm.nih.gov/nuccore/CM036749.1) | SCAF_21 SCAF_98 |
| Z | [CM036750.1](https://www.ncbi.nlm.nih.gov/nuccore/CM036750.1) | SCAF_4 |

**Supplementary Table 2**. Basic assembly statistics from NCBI for all genomes currently available in the bird family Icteridae. The primary (bAgeTri1.0.p) and alternate (bAgeTri1.0.a) assemblies for tricolored blackbird presented in this paper are bolded.

|  | *Agelaius tricolor* |  |  | *Agelaius phoeniceus* |  | *Molothrus ater* |  | *Quiscalus mexicanus* |
| --- | --- | --- | --- | --- | --- | --- | --- | --- |
| NCBI summary statistic | **bAgeTri1.0.p** | **bAgeTri1.0.a** | TRBL-genome | ASM1339853v1 | ASM2074582v1 | BPBGC_Mater_1.0 | ASM1340115v1 | ASM1339903v1 |
| Total sequence length | **1,157,608,456** | **1,142,379,013** | 1,075,086,955 | 1,086,309,140 | 1,188,529,604 | 1,087,312,585 | 1,229,058,992 | 1,041,398,572 |
| Total ungapped length | **1,157,598,756** | **1,142,369,313** | 1,074,882,755 | 1,059,786,890 | 1,188,478,804 | 1,087,156,585 | 1,200,298,508 | 1,036,029,931 |
| Gaps between scaffolds | **0** | **0** | 0 | 0 | 23 | 34 | 0 | 0 |
| Number of scaffolds | **214** | **450** | 70,522 | 42,265 | 440 | 447 | 144,198 | 56,227 |
| Scaffold N50 | **47,739,980** | **63,409,879** | 103,912 | 1,693,451 | 36,435,137 | 52,124,711 | 1,946,625 | 93,215 |
| Scaffold L50 | **9** | **6** | 2,648 | 166 | 8 | 7 | 170 | 2,950 |
| Number of contigs | **311** | **547** | 72,564 | 75,155 | 515 | 691 | 196,774 | 79,281 |
| Contig N50 | **22,601,135** | **21,939,630** | 87,741 | 78,381 | 22,833,505 | 13,273,966 | 89,160 | 60,532 |
| Contig L50 | **13** | **14** | 3,213 | 3,691 | 13 | 25 | 3,653 | 4,536 |
| Total number of chromosomes and plasmids | **1** | **0** | 0 | 0 | 37 | 34 | 0 | 0 |
| Number of component sequences (WGS or clone) | **214** | **450** | 70,522 | 42,265 | 515 | 691 | 144,198 | 56,227 |
| Coverage | **41x** | **41x** | 100x | 120x | 38x | 200x | 63x | 54x |
