# Supplementary material for: A reference genome assembly of the declining tricolored blackbird, Agelaius tricolor
Source: J Hered. 2022 Sep 13;114(1):44–51. doi: 10.1093/jhered/esac053 (PMC10019024; doi:10.1093/jhered/esac053)
Supplement: esac053_suppl_Supplementary_Figure_S1 [file esac053_suppl_supplementary_figure_s1.pdf]

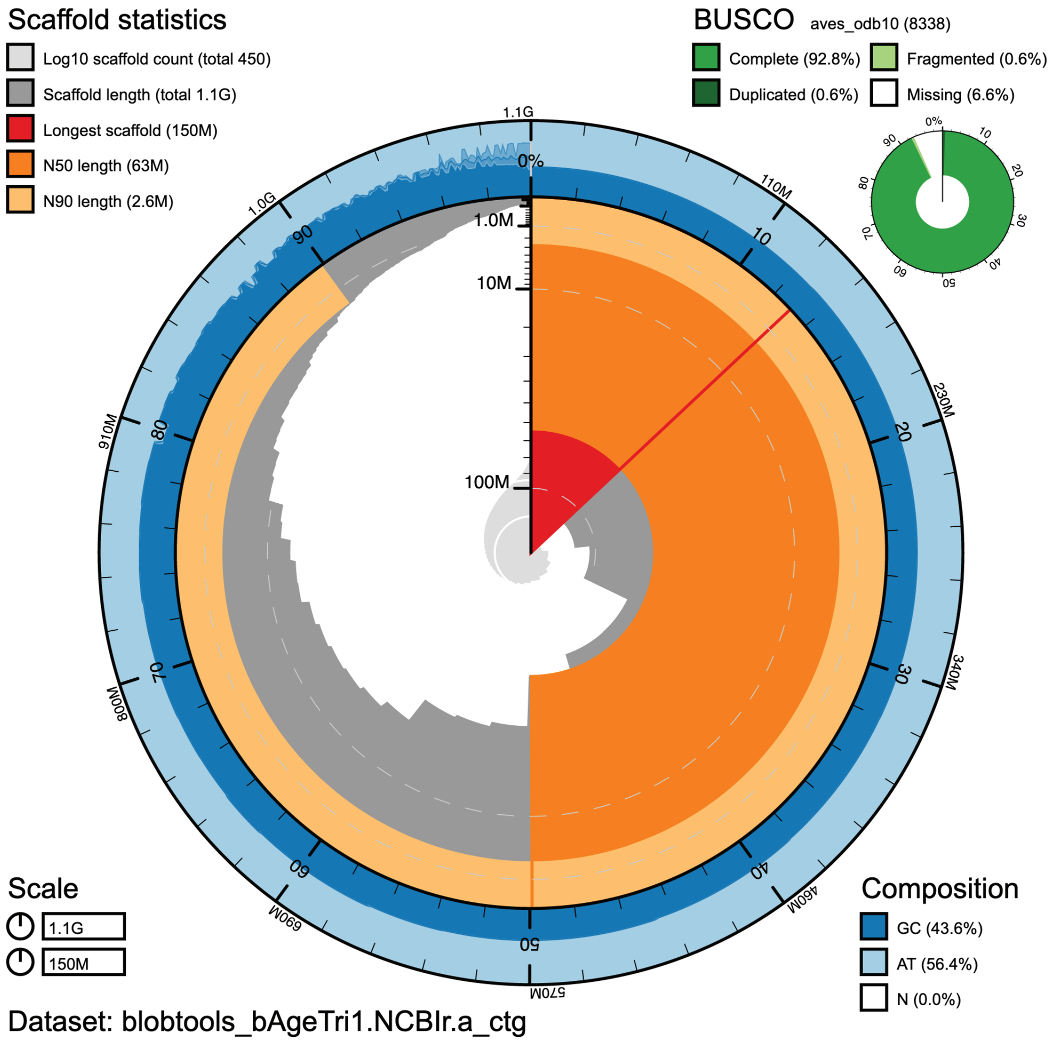

Supplementary Figure 1. BlobToolKit Snail plot of tricolored blackbird alternate genome assembly (bAgeTri1.0.a). The plot circle represents the full size of the assembly. From the inside-out, the central plot covers length-related metrics. The red line represents the size of the longest scaffold; all other scaffolds are arranged in size order moving clockwise around the plot and drawn in gray starting from the outside of the central plot. Dark and light orange arcs show the scaffold N50 and scaffold N90 values. The central light gray spiral shows the cumulative scaffold count with a white line at each order of magnitude. White regions in this area reflect the proportion of Ns in the assembly. The dark vs. light blue area around it shows mean, maximum and minimum GC vs. AT content at 0.1% intervals (Challis et al. 2020).
